# Tea Making

**Published:** 1890-06

**Authors:** 


### TEA MAKING.

In preparing tea, the water to be used should never be poured directly from the kitchen kettle into the urn. It should be cold, *fresh* water, brought absolutely to the boiling point. The tea used will, of course, differ according to taste, but none is better for the purpose than the best English breakfast. The leaves must be placed in the pot in the proportion of a heaping teaspoonful to each person. Upon these leaves pour a small quantity of boiling water ; never use all of the latter needed at once, as a sudden rush will certainly "drown" the tea. Now pull the cozy over the tea-pot and allow the contents to draw a few moments, when you will have the best infusion possible ; repeat this process as many times as needed ; after using the first potful and filling once more with boiling water, the tea loses its strength and flavor. Boiled tea is hurtful and breakfast tea should never be steeped upon the stove. It will not often be necessary to strain where these directions are followed, but the sudden addition of water floats the leaves which do not again settle.

